# A Bifunctional SARS-CoV-2 Entry Inhibitor Targeting the Host Protease TMPRSS2 and Viral Spike Protein HR1 Region

**DOI:** 10.3390/ijms26178289

**Published:** 2025-08-26

**Authors:** Huan Wang, Qing Li, Zhe Yin, Shu Du, Longbo Zheng, Xinmeng Du, Anqi Shi, Jichun Li, Weiguo Shi, Fei Yu, Junhai Xiao, Chao Wang

**Affiliations:** 1 State Key Laboratory of National Security Specially Needed Medicines, Beijing Institute of Pharmacology and Toxicology, Beijing 100850, China; 2 Hebei Key Laboratory of Analysis and Control of Zoonotic Pathogenic Microorganism, College of Life Sciences, Hebei Agricultural University, Baoding 071001, China; 3 Key Laboratory of Structure-Based Drug Design & Discovery of the Ministry of Education, Shenyang Pharmaceutical University, Shenyang 110016, China; 4 College of Pharmacy, Mudanjiang Medical University, Mudanjiang 157000, China

**Keywords:** SARS-CoV-2, TMPRSS2, 6-HB, entry inhibitors, multi-target-directed ligands

## Abstract

SARS-CoV-2 entry into host cells involves multiple steps and is a highly orchestrated process. Both the host protease TMPRSS2 and the HR1/HR2 segment within the spike (S) protein play a crucial role in promoting viral invasion. Herein, we report a series of bifunctional SARS-CoV-2 entry inhibitors formed by covalently linking a TMPRSS2 inhibitor, Camostat (Cm), and an HR1-targeting peptide fusion inhibitor IPB19 via a poly (ethylene glycol) (PEG) linker. Among them, IP4X and IP4Z display potent inhibitory activities against SARS-CoV-2 with similar IC_50_ values of 0.16 μM and 0.17 μM, respectively. The efficacy surpassed that of their parent inhibitors by approximately 28-fold relative to Camostat and 15-fold relative to IPB19. We confirm that IP4X and IP4Z exhibit a dual-targeting mechanism by binding to both TMPRSS2 and HR1 region of S protein. These findings highlight the potential of the bifunctional inhibitors for further development as a novel multitarget therapy against SARS-CoV-2 infection and enrich the understanding of S-mediated entry of SARS-CoV-2 into host cells.

## 1. Introduction

The global pandemic of coronavirus disease 2019 (COVID-19), caused by the severe acute respiratory syndrome coronavirus 2 (SARS-CoV-2), has recently resulted in more than 800 million confirmed cases, with about 6.98 million deaths worldwide [1]. During and after the COVID-19 pandemic, the extensive global dissemination of SARS-CoV-2 and its potent human-to-human transmission were alarming. As a positive-sense single-stranded RNA virus, SARS-CoV-2 has a high mutation rate, as it lacks the error correction mechanism of their genetic material and, therefore, can evolve to become novel variants with limited sensitivity to existing antivirals and reduced potency of vaccines through immune escape, thereby triggering subsequent waves of infection [2,3]. The ongoing spread of SARS-CoV-2 and the emergence of variants keep threatening human life and health, highlighting the urgent need to develop antiviral drugs that possess new mechanism of action.

**Figure 1:**
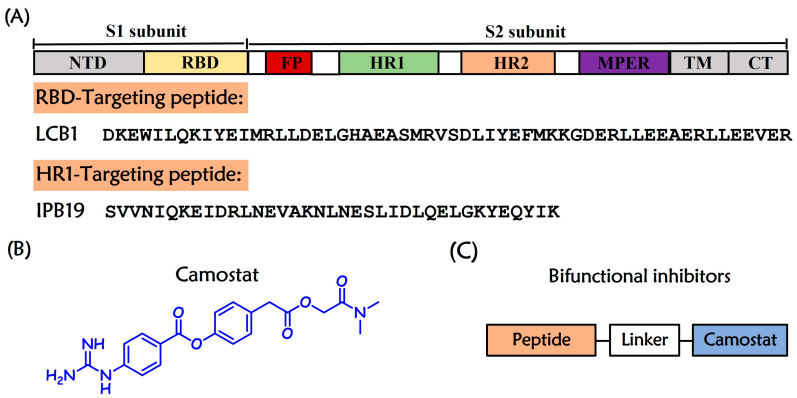
Schematic illustration of SARS-CoV-2 S protein and entry inhibitors. (**A**) Schematic representation of the primary structure of SARS-CoV-2 envelope glycoprotein S1 subunit and S2 subunit and peptides targeting RBD or HR1 regions. NTD, N-terminal domain; RBD, receptor-binding domain; FP, fusion peptide; HR1, heptad repeat 1; HR2, heptad repeat 2; MPER, membrane-proximal external region; TM, transmembrane region; CT, cytoplasmic tail. (**B**) Chemical structure of the TMPRSS2 inhibitor Camostat. (**C**) Design framework of bifunctional inhibitors.

Viral entry is the first step of infection and the key target for therapeutics [4]. This process is executed by the spike (S) protein on the envelope of SARS-CoV-2, which is composed of the S1 subunit and the S2 subunit, which form trimers of the S1/S2 heterodimer [5,6]. Initially, the S1 subunit binds to the host cell receptor angiotensin-converting enzyme 2 (ACE2) via the receptor-binding domain (RBD) [7,8]. This triggers conformational changes in the S protein, leading to the exposure of the S2 subunit and cleavage by host proteases. Transmembrane protease serine 2 (TMPRSS2) plays a pivotal role in this cleavage event, which is essential for initiating the membrane fusion process of the S2 subunit [9,10]. Subsequently, the fusion peptides (FPs) on the S2 subunit were exposed and inserted into the target cell membrane. Following that, heptad repeat 1 (HR1) and heptad repeat 2 (HR2) assemble into a six-helix bundle (6-HB) structure that pulls together the host and virus membranes, resulting in fusion between the two membranes, which ultimately will lead to the release of the viral core into the cell (Figure 1A) [11,12]. The host factors and viral factors involved in the virus entry process have been attractive antiviral targets for the development of anti-SARS-CoV-2 therapy. For example, using a de novo computational design strategy, Cao and coworkers developed a 56-amino acid miniprotein termed LCB1, showing highly effective antiviral activity. LCB1 exhibited strong binding affinity to the RBD, thus blocking the interaction between the S protein and ACE2 [13]. Additionally, the small molecule Camostat (Cm) was demonstrated to block SARS-CoV-2 entry into host cells by binding to the active site of TMPRSS2, which reduces its protease activity and prevents cleavage of the S2 subunit (Figure 1B) [14,15,16]. Also, the HR1 and HR2 regions are highly conserved in the S protein. Therefore, targeting the HR1-HR2 interaction to block the formation of the fusogenic 6-HB core structure represents a rational approach to inhibiting SARS-CoV-2 infection [8,17,18]. Yu et al. utilized the HR2 region of SARS-CoV-2 as the parental scaffold and identified the HR1-targeting fusion inhibitor peptide IPB19, which exhibited inhibitory activity against SARS-CoV-2 [19].

Combination therapy, which involves the use of two or more antiviral drugs targeting different aspects of the viral life cycle, has been proven useful in treating viral infectious diseases [20]. Compared with individual antiviral agents, combination therapy has achieved significant success in enhancing antiviral efficacy and reducing drug resistance. However, medicine combination brings about new concerns, like drug–drug interactions, pharmacokinetic/pharmacodynamic complexity, and cumulative toxicities [21,22]. In this context, the strategy of designing multitarget-directed ligands (MTDLs) is attracting more attention due to their reliable efficacy and safety [23]. The MTDLs containing two or more activity-related pharmacophores into a single entity, have improved therapeutic efficacy and are not involved in complex drug–drug interactions [23,24].

RBD-targeting miniprotein LCB1, TMPRSS2 inhibitor Camostat and HR1-targeting fusion inhibitor IPB19 have been demonstrated to possess anti-SARS-CoV-2 activity through blocking the process of viral entry into host cells. In this study, events in the process of SARS-CoV-2 invasion are selected as targets for our dual-target inhibitor design (Figure 1C), considering that SARS-CoV-2 entry involves multiple steps and is a highly orchestrated process that offers multiple druggable targets. We describe the discovery of a series of bispecific inhibitors, which are generated by the small-molecule TMPRSS2 inhibitor Camostat and the fusion inhibitor peptide IPB19 via a poly (ethylene glycol) (PEG) spacer. Notably, compounds IP4X and IP4Z displayed markedly increased anti-SARS-CoV-2 potency compared to parent inhibitors, with enhancements of approximately 28-fold relative to Camostat and 15-fold relative to IPB19. Furthermore, we found that IP4X and IP4Z exhibited a bifunctional mechanism by targeting both the TMPRSS2 and HR1 regions of S protein through multiple biophysical and functional approaches. Therefore, our study not only provides a potent dual-target entry inhibitor lead molecule for novel anti-SARS-CoV-2 drug development but also enriches the understanding of S-mediated entry of SARS-CoV-2 into host cells.

## 2. Results and Discussion

### 2.1. Design and Chemistry

**Figure 2:**
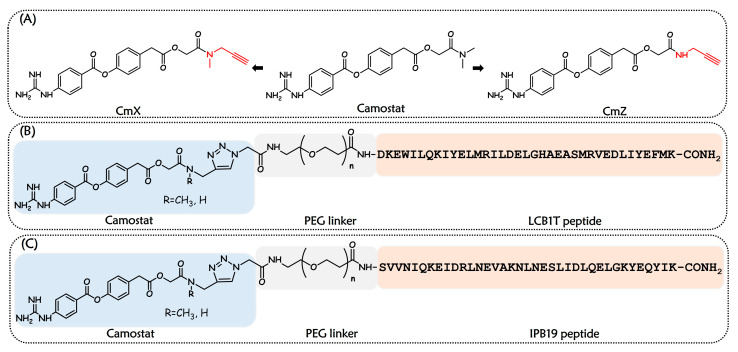
Design strategy of dual-target ligands. (**A**) Camostat and its analogs for chimera construction. Conjugation points are highlighted in red. The structure of (**B**) Class I chimeras and (**C**) Class II chimeras. The Camostat component is highlighted with blue shading, the peptides are highlighted with orange shading, and the PEG linker is highlighted with gray shading. The letter “n” represents the number of PEG units in the linker.

SARS-CoV-2 entry initiates with RBD-ACE2 interaction, followed by TMPRSS2-mediated S protein cleavage, which triggers 6-HB formation between HR1 and HR2, leading to membrane fusion and infection. We designed two classes of bispecific entry inhibitors against SARS-CoV-2. Class I compounds targeting RBD and TMPRSS2 to prevent the initial attachment and Class II compounds inhibiting TMPRSS2 and HR1 to block downstream fusion. Specifically, we first chose to use Camostat as the TMPRSS2 inhibitor pharmacophore. As a clinically available drug approved in Japan (as mesylate salt) to treat pancreatitis, Camostat inhibits TMPRSS2 in vitro and has been in a clinical trial for the treatment of COVID-19 [14,16,25]. Its X-ray crystal structure, combined with theoretical calculations, shows that the guanidine group of Camostat plays a critical role in interacting with the binding pocket of TMPRSS2 [14,26]. To minimize potential disruption of the original interactions between Camostat and TMPRSS2 possibly caused by the introduced fusion inhibitor pharmacophore, the terminal dimethylformyl group on the right side of Camostat was selected as the attachment site. We replaced the dimethylformyl group with an N-methyl-2-propynylcarboxamide and 2-propynylcarboxamide moiety, respectively, yielding Camostat derivatives CmX and CmZ (Figure 2A). Subsequently, a truncated LCB1 derivative, named LCB1T, served as the RBD-binding moiety for covalent conjugation to Camostat analogs via PEG linkers, yielding the bifunctional chimeras (Class I) (Figure 2B). In parallel, IPB19, as the HR1-targeting fusion inhibitor, similarly was coupled to Camostat analogs through PEG spacers to generate the other bifunctional compounds (Class II) (Figure 2C).

**Scheme 1:**
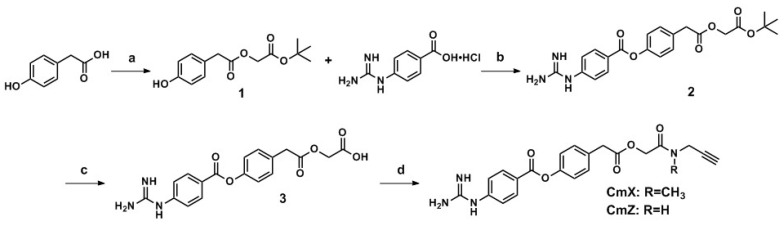
Synthesis of CmX and CmZ. Reagents and conditions: (a) Et3N, MeCN, 4-Hydroxyphenylacetic acid, 0 °C to 83 °C reflux; (b) DCC, Pyridine, 0 °C to r.t.; (c) TFA, DCM; (d) HATU, DIEA, N-methylpropargylamine or Propynylamine, r.t.

The synthetic pathways to CmX and CmZ are illustrated in Scheme 1. Initially, 4-Hydroxyphenylacetic acid was coupled with tert-Butyl bromoacetate in the presence of triethylamine (Et3N) and acetonitrile (MeCN) under reflux conditions to yield the active ester intermediate **1**. Following this, compound **2** was obtained from **1** by esterification with 4-guanidinobenzoic acid hydrochloride. By saponification, **2** was converted into carboxylic acid intermediates **3**. Finally, the synthesis of the Camostat derivatives CmX and CmZ was accomplished by amination of **3** with N-methylpropargylamine and propargylamine, respectively. The hybrid molecules were synthesized via solid-phase peptide synthesis (SPPS) for peptide preparation, followed by conjugation of Camostat analogs to the N-terminus of peptides through copper(I)-catalyzed alkyne-azide cycloaddition (CuAAC) reaction [27,28,29].

### 2.2. Bifunctional Entry Inhibitors Demonstrated Excellent Anti-SARS-CoV-2 Activity

First, the inhibitory activity of Class I and Class II chimeras against wild-type SARS-CoV-2 pseudovirus (PsV) infection on Caco2 cells was measured. As shown in Table 1, we sequentially conjugated diverse PEG linkers and Camostat derivatives to the N-terminus of LCB1T, thereby resulting in a series of chimeric molecules from LP4X to LP24Z. They maintained potent antiviral activity comparable to LCB1T, with IC_50_ values ranging from 0.32 to 0.40 μM. In comparison, the Class II compounds, obtained by covalently linking Camostat analogs to the IPB19 via a PEG linker, exhibited dramatically increased antiviral potency. In particular, IP4X had an IC_50_ value of 0.16 μM, which was 15-fold and 28-fold greater than that of IPB19 and Camostat alone, respectively. The observed antiviral profile of Class I chimeras appeared to be primarily driven by LCB1T, with minimal contribution from the Camostat component. As LCB1T demonstrated superior antiviral potency relative to Camostat, we speculate that this phenomenon may be attributed to the differences in the potencies of constituent ligands LCB1T and Camostat against their respective targets, resulting in the pharmacological activity of Camostat being overshadowed by LCB1T [14,30]. This shows that achieving balanced and multiselective potency toward multiple targets is critical and challenging [30,31]. We also observed that IP4Z with the CmZ possessed inhibitory activity similar to that of IP4X, indicating removal of the methyl at the terminal amide position of the right side of Camostat has no significant impact on antiviral activity. In addition, IP24X and IP24Z were generated by substituting the PEG4 linker in IP4X and IP4Z with a PEG24 linker, respectively. Disappointingly, the replacement of PEG4 by PEG24 led to a slight decrease in antiviral activity, suggesting that the linker PEG4 might adequately bridge both binding sites and place two pharmacophores in the right position. The ongoing spread of SARS-CoV-2 with evolutionary mutations leads to the emergence of many variants with limited sensitivity to existing antivirals and reduced potency of vaccines through immune escape. Among divergent variants, Omicron emerged with the largest number of mutations and has now dominated the worldwide pandemic. Therefore, we evaluated antiviral activity of IP4X and IP4Z against Omicron. In the Caco2 cells, IP4X and IP4Z inhibited the entry of pseudotyped SARS-CoV-2 Omicron strains with IC_50_ values of 0.83 μM and 0.76 μM, respectively (Table S3).

### 2.3. Bifunctional Entry Inhibitors Could Interact with HR1 Region of SARS-CoV-2 S Protein

**Figure 3:**
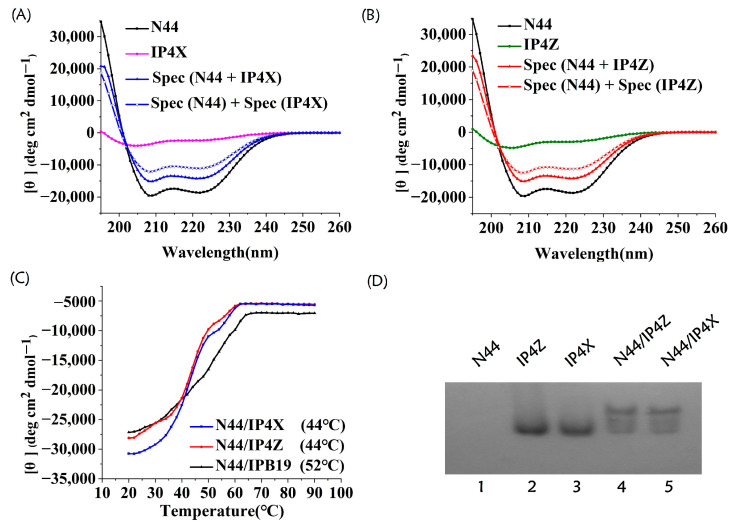
Interaction of IP4X and IP4Z with SARS-CoV-2 HR1-derived target mimic peptide N44. The α-helicity of the (**A**) N44/IP4X and (**B**) N44/IP4Z complexes was determined by CD spectroscopy, with the final concentration of each peptide being 10 μM in PBS. [Spec (N44 + chimera), solid symbols] describes the CD spectra of peptide mixtures. In contrast, [Spec (N44) + Spec (chimera), open symbols] describes the sum of the spectra of the related isolated peptides. (**C**) Thermostability of N44/IP4X, N44/IP4Z, and N44/IPB19 complexes. *Tm* values are shown in parentheses. The final concentration of each peptide in PBS was 10 μM. (**D**) The interactions between IP4X and IP4Z with N44 were visualized by N-PAGE. Lane 1, N44; lane 2, IP4Z; lane 3, IP4X; lane 4, N44 + IP4Z; lane 5, N44 + IP4X. Each peptide was used at a final concentration of 100 μM.

To investigate how the bifunctional entry inhibitors interacted with the targets, we conducted multiple biophysical and functional approaches to understand the mechanism of action of these chimeras. IP4X and IP4Z were selected as representatives of chimeras for interaction analysis due to their best results in anti-SARS-CoV-2 activity. The circular dichroism (CD) spectroscopy was first applied to determine whether IP4X and IP4Z could bind to the SARS-CoV-2 HR1-derived target mimic peptide N44. As shown in Figure 3A, IP4X alone displayed no or minor α-helicity, and N44 alone displayed 57% α-helicity. However, an equimolar mixture of the two peptides exhibited a total helical content of 43% for the system, which exceeded the sum of the component spectra. This implies a considerable conformational change due to the interaction between N44 and IP4X. In a similar manner, it was observed that the α-helicity of the N44/IP4Z mixture was markedly elevated in comparison to the mathematical sum of the CD signals from the individual peptides (Figure 3B). An interaction between bifunctional inhibitors and the target surrogate peptide is implied by these results, leading to a change in helical structures in both the component inhibitors and N44, as would be expected for the formation of α-helical complexes [32,33]. Additionally, both the N44/IP4X and N44/IP4Z complex showed good thermal stability, with a *Tm* of 44 °C. The thermal stability of the N44/IPB19 complex was also determined as a control, with a *Tm* value of 52 °C (Figure 3C). Subsequently, native polyacrylamide gel electrophoresis (N-PAGE) analysis was employed to observe the complexes formed between the bifunctional entry inhibitors and target peptide N44 (Figure 3D). N44 showed no band in the native gel because it carried net positive charges and could migrate up and off the gel, but the negatively charged peptides IP4X and IP4Z showed specific bands. Upon mixing IP4X or IP4Z with N44, a new band corresponding to the 6-HB appeared. This observation aligns with our findings from the CD studies and indicates that bifunctional inhibitors are capable of targeting the SARS-CoV-2 HR1 region and interacting with the HR1-derived peptide N44 to form stable complexes.

### 2.4. Bifunctional Entry Inhibitors Could Bind to Host Protease TMPRSS2

**Figure 4:**
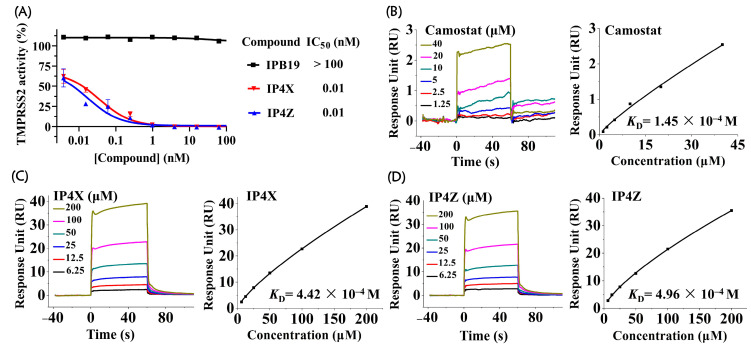
Interaction of IP4X and IP4Z with TMPRSS2. (**A**) TMPRSS2 inhibition by chimeric inhibitors and peptide IPB19. Experiments were conducted with 5 nM recombinant TMPRSS2 and 100 µM Boc-QAR-AMC peptide substrate, and the data are expressed as means ± the standard deviation (error bar) for assays performed in two technical replicates. IC_50_ values were calculated with GraphPad Prism 8 software. SPR analysis of (**B**) Camostat, (**C**) IP4X, and (**D**) IP4Z with TMPRSS2. *K_D_* values were determined using Biacore 8K evaluation software version 1.

We next asked whether the bifunctional design might include TMPRSS2 as one of the antiviral targets. To evaluate this question, we conducted a biochemical assay using active TMPRSS2 protease and a fluorogenic peptide substrate to confirm that the chimeric compounds inhibit the protease activity of recombinant TMPRSS2 [34]. As shown in Figure 4A, at a concentration up to 100 nM, IPB19, as a negative control, no inhibition of protease activity of TMPRSS2 was detected. Notably, IP4X and IP4Z exhibited inhibitory activity against TMPRSS2 at an IC_50_ of 0.01 nM. Subsequently, we determined the binding affinity of the molecular interaction between two dual-target inhibitors and TMPRSS2 by performing surface plasmon resonance (SPR) analysis. The SPR analysis revealed direct interaction between two entry inhibitors and TMPRSS2, with *K_D_* values of 4.96 × 10^−4^ M and 4.42 × 10^−4^ M, respectively (Figure 4C,D). As a control, Camostat exhibited a binding affinity to TMPRSS2 with a *K_D_* value of 1.45 × 10^−4^ M (Figure 4B), which is comparable to that of IP4X and IP4Z. These results support the notion that the chimeric inhibitors could target the TMPRSS2 and suppress the protease activity of TMPRSS2. We observed distinct values between the IC_50_ values of bifunctional inhibitors against TMPRSS2 and their *K_D_* values for TMPRSS2. This inconsistency is related to the mechanism of Camostat as a covalent inhibitor of TMPRSS2. Initially, Camostat binds to TMPRSS2, forming a noncovalent precomplex. The ester group in guanidinobenzoyl moiety of Camostat then reacts with the catalytic serine residue of TMPRSS2 to form the covalent acyl-enzyme complex and disables the protease activity. The IC_50_ values are primarily determined by the long-lived covalent complex formation, while the *K_D_* values solely reflect the metastable process of noncovalent complex formation. Therefore, the observed discrepancy between these two parameters is mechanistically well rationalized [14,26].

## 3. Materials and Methods

### 3.1. Chemistry

All reagents were purchased from commercial suppliers and utilized directly without additional purification unless otherwise indicated. Reaction progress was tracked via thin-layer chromatography (TLC) on silica gel GF254 plates from the Qingdao Haiyang Chemical Company (Qingdao, China). Purification was achieved through column chromatography using silica gel (200–300 mesh) sourced from the Qingdao Haiyang Chemical Company. Nuclear magnetic resonance (NMR) spectra, including ^1^H (400 MHz) and ^13^C (100 MHz), were acquired on a Quantum-I plus AS400 spectrometer (Zhongke-Niujin Ltd. Wuhan, China) employing tetramethylsilane (TMS) as the internal reference. Chemical shifts were denoted in *δ* (ppm), and coupling constants (*J*) were presented in hertz (Hz). Mass spectra (MS) for small molecules were obtained using a 1260/G6230A liquid chromatography–mass spectrometer equipped with an electrospray ionization (ESI) source from Agilent Inc (Santa Clara, CA, USA). Purity analysis of compounds intended for biological assays was conducted on an analytical reversed-phase high-performance liquid chromatography (RP-HPLC) system (Shimadzu, Kyoto, Japan) fitted with a Waters X-Bridge C8 column (4.6 × 250 mm, 5 μm). The purity of all final compounds was confirmed to be ≥95%. The molecular weight of peptides was determined by matrix-assisted laser desorption ionization-time-of-flight mass spectrometry (MALDI-TOF MS) using an Ultraflex instrument from Bruker Daltonics Inc (Billerica, MA, USA).

### 3.2. General Protocol for the Synthesis of Small-Molecule Compounds

**Synthesis of Compound 1.** A mixture of 20 g (131.44 mmol) of 4-hydroxyphenylacetic acid and 30.6 g (157.73 mmol) of t-butyl bromoacetate was placed in a reaction flask. The mixture was dissolved in 320 mL of acetonitrile under stirring. Subsequently, 21.9 mL (157.73 mmol) of triethylamine was added dropwise to the solution while stirring at 0 °C in an ice bath. After the addition was complete, the reaction mixture was heated to reflux at 83 °C and maintained for 8 h. Upon completion of the reaction, the mixture was cooled to room temperature, and the pH was adjusted to approximately 7 using 1N HCl. The solution was then extracted three times with ethyl acetate (EA). The combined organic layers were washed three times with saturated sodium chloride solution, dried over anhydrous sodium sulfate, and concentrated under reduced pressure. The crude product was purified by column chromatography to supply compound **1** as a white solid (23.74 g, 67.83%). ^1^H NMR (600 MHz, DMSO-*d*_6_) *δ* 9.31 (s, 1H), 7.07 (d, *J* = 8.5 Hz, 2H), 6.70 (d, *J* = 8.4 Hz, 2H), 4.52 (s, 2H), 3.60 (s, 2H), 1.38 (s, 9H). ^13^C NMR (151 MHz, DMSO-*d*_6_) *δ* 171.13, 166.72, 156.31, 130.36, 123.97, 115.13, 81.52, 61.22, 39.02, 27.62. MS (*m*/*z*): calculated for C_14_H_18_O_5_, 266.12. LC-MS (*m*/*z* (rel intens)): 289.11 (M + Na).

**Synthesis of Compound 2. Step 1.** Under nitrogen protection, 13.36 g (61.95 mmol) of 4-guanidinobenzoic acid hydrochloride was added to a reaction flask and dissolved in 650 mL of anhydrous pyridine. The solution was cooled in an ice bath, and then 13.94 g (67.58 mmol) of *N,N*′-dicyclohexylcarbodiimide (DCC) was added. The mixture was stirred for 30 min at 0 °C, then the ice bath was removed, and the reaction was continued at room temperature for an additional 1 h. **Step 2.** Subsequently, 15 g (56.32 mmol) of **1** was added to the reaction mixture, and the stirring was continued for 4 h. The reaction mixture was filtered, and the precipitate was washed multiple times with small portions of anhydrous pyridine. The filtrate was collected and concentrated under reduced pressure. A small amount of acetone was added to remove residual pyridine solvent. The mixture was then diluted with anhydrous ether, filtered again, and the filtrate was collected and allowed to dry by evaporation. The crude product was purified by column chromatography to supply compound **2** as a yellow solid (7.72 g, 32.1%). ^1^H NMR (600 MHz, DMSO-*d*_6_) *δ* 8.16 (d, *J* = 8.7 Hz, 2H), 7.94 (s, 4H), 7.41 (dd, *J* = 28.8, 8.6 Hz, 4H), 7.24 (d, *J* = 8.5 Hz, 2H), 4.57 (s, 2H), 3.82 (s, 2H), 1.40 (s, 9H). ^13^C NMR (151 MHz, DMSO-d6) *δ* 171.15, 167.12, 164.47, 156.20, 149.97, 141.70, 132.23, 131.91, 131.09, 125.79, 123.10, 122.24, 82.08, 61.85, 49.05, 28.11. MS (*m*/*z*): calculated for C_22_H_25_N_3_O_6_, 427.17. LC-MS (*m*/*z* (rel intens)): 428.18 (M + H).

**Synthesis of Compound 3.** Compound **2** (7.2 g, 16.85 mmol) was weighed and placed into a reaction flask, dissolved by stirring in 200 mL of dichloromethane (DCM), followed by the addition of 100 mL of trifluoroacetic acid (TFA). The solution was clear and stirred at room temperature for 2.5 h. The reaction mixture was then concentrated under reduced pressure and purified by lyophilization to remove TFA. The crude product was purified by column chromatography to supply compound **3** as a white solid (9.5 g, 91.2%). ^1^H NMR (600 MHz, DMSO-*d*_6_) *δ* 13.09 (s, 1H), 8.14–8.18 (m, 2H), 7.88 (s, 4H), 7.37–7.46 (m, 4H), 7.21–7.26 (m, 2H), 4.60 (s, 2H), 3.83 (s, 2H). ^13^C NMR (151 MHz, Methanol-*d*_4_), *δ* 171.33, 169.99, 164.32, 156.33, 150.01, 140.45, 131.89, 131.51, 130.36, 127.44, 123.54, 121.32, 60.64, 39.22. MS (*m*/*z*): calculated for C_18_H_17_N_3_O_6_, 371.11. LC-MS (*m*/*z* (rel intens)): 372.12 (M + H).

**Synthesis of Compound CmX.** Compound **3** (5 g, 13.47 mmol) was weighed and placed into a reaction flask, dissolved by stirring in 80 mL of DMF. Subsequently, 1.92 g (14.77 mmol) of DIEA and 5.64 g (14.77 mmol) of HATU were added. The mixture was stirred at room temperature for 2 h, after which 1.02 g (13.47 mmol) of N-methylpropargylamine was added and stirred at room temperature for an additional 18 h. The reaction mixture was then diluted with 80 mL of water and extracted three times with EA. The combined organic layers were washed three times with saturated sodium chloride solution, dried over anhydrous sodium sulfate, and concentrated. The crude product was purified by column chromatography to supply compound **CmX** as a yellow oil (3.06 g, 55.8%). ^1^H NMR (600 MHz, Acetone-*d*_6_) *δ* 8.26 (d, *J* = 8.5 Hz, 2H), 7.64 (d, *J* = 8.5 Hz, 2H), 7.56 (s, 2H), 7.46 (dd, *J* = 11.1, 8.6 Hz, 2H), 7.24 (t, *J* = 8.6 Hz, 2H), 4.66–4.97 (m, 2H), 4.15–4.53 (m, 2H), 3.78–3.91 (m, 2H), 3.65–3.78 (m, 1H), 2.93–3.11 (m, 3H), 2.76 (d, *J* = 27.2 Hz, 1H). ^13^C NMR (151 MHz, Methanol-*d*_4_) *δ* 171.39, 167.41, 164.31, 156.33, 150.01, 140.44, 131.94, 131.51, 130.42, 123.54, 121.31, 77.51, 72.18, 61.33, 39.23, 35.88, 32.08. MS (*m*/*z*): calculated for C_22_H_22_N_4_O_5_, 422.16. LC-MS (*m*/*z* (rel intens)): 423.17 (M + H).

**Synthesis of Compound CmZ.** Compound **3** (3 g, 8.08 mmol) was placed in a reaction flask and dissolved by stirring in 60 mL of *N,N*-dimethylformamide (DMF). Subsequently, 3.38 g (8.89 mmol) of *N,N*-diisopropylethylamine (DIEA) and 1.15 g (8.89 mmol) of 2-(7-azabenzotriazol-1-yl)-*N,N,N*′*,N*′-tetramethyluronium hexafluorophosphate (HATU) were added. The mixture was stirred at room temperature for 2 h, after which 0.49 g (8.08 mmol) of propargylamine was added and stirred at room temperature for an additional 18 h. The reaction mixture was then diluted with 60 mL of water and extracted three times with EA. The combined organic layers were washed three times with saturated sodium chloride solution, dried over anhydrous sodium sulfate, and concentrated. The crude product was purified by column chromatography to supply compound **CmZ** as a yellow oil (1.93 g, 58.4%). ^1^H NMR (600 MHz, DMSO-*d*_6_) *δ* 8.52 (t, *J* = 5.4 Hz, 1H), 8.16 (d, *J* = 8.7 Hz, 2H), 7.69 (s, 4H), 7.41 (dd, *J* = 18.8, 8.6 Hz, 4H), 7.23 (d, *J* = 8.5 Hz, 2H), 4.53 (s, 2H), 3.90 (dd, *J* = 5.5, 2.5 Hz, 2H), 3.84 (s, 2H), 3.14 (t, *J* = 2.5 Hz, 1H). ^13^C NMR (151 MHz, Methanol-*d*_4_) *δ* 171.07, 168.25, 164.33, 156.31, 150.05, 140.41, 131.86, 131.53, 130.38, 123.58, 121.38, 78.85, 70.93, 62.26, 39.15, 27.84. MS (*m*/*z*): calculated for C_21_H_20_N_4_O_5_, 408.14. LC-MS (*m*/*z* (rel intens)): 409.16 (M + H).

### 3.3. Peptide Synthesis

Peptides were synthesized using a standard solid-phase Fmoc (fluorenylmethoxycarbonyl) strategy with a CS biopolypeptide synthesizer on Rink amide resin, which had a loading capacity of 0.53 mmol/g. The amino acids were coupled using O-benzotriazol-1-yl-*N,N,N*′*,N*′-tetramethyluronium hexafluorophosphate (HBTU) (3 equiv) and DIEA (6 equiv) as the activator and base, respectively, in DMF. The Fmoc protecting group was removed with a 20% piperidine solution in DMF. For peptides containing a deprotected lysine residue at the C-terminus, a specialized deprotection procedure was employed, consisting of four 3 min washes with 2% hydrazine hydrate in DMF. This step facilitated the conjugation of 2-azidoacetic acid, which was achieved by adding 3 equiv of 2-azidoacetic acid, 3 equiv of HBTU, and 6 equiv of DIEA in DMF to the resin, followed by stirring for 2 h. The peptides were then cleaved from the resin and deprotected using reagent K, which comprised 82.5% TFA, 5% anisole, 5% *m*-cresol, 5% water, and 2.5% ethanedithiol. The crude peptides were precipitated with cold diethyl ether and subsequently lyophilized. Purification of the crude peptide products was carried out using RP-HPLC (Shimadzu preparative HPLC system), and the purity of each peptide was verified to be ≥95% by analytical RP-HPLC (Shimadzu analytical HPLC system) (Figure S1). HPLC method used for the purification of peptide compounds and the analysis of peptide compounds are provided in Table S1 and Table S2, respectively.

### 3.4. Synthesis of Bifunctional Chimeras

The purified azide-modified peptide inhibitors and terminal alkyne-modified small-molecule inhibitors were conjugated using a CuAAC reaction. The catalytic system consisted of copper(II) sulfate pentahydrate (CuSO_4_·5H_2_O) and the reducing agent sodium L-ascorbate, with a solvent mixture of tert-butanol and dd H_2_O. For each reaction, 10 mg of the azide-modified peptide (purity > 95%) and 2.4 equiv of the terminal alkyne-modified small molecule were weighed out. Each component was dissolved in 3 mL of a tert-butanol/dd H_2_O solution (*v*/*v* = 1/1) and then mixed together. Separately, 10 equiv of CuSO_4_·5H_2_O and 50 equiv of sodium L-ascorbate were dissolved in 4 mL of dd H_2_O to form a yellow solution, which was then added to the mixture of the peptide and small molecule. After vortexing at room temperature for 5 min, the reaction progress was monitored by RP-HPLC until completion.

### 3.5. Inhibition of SARS-CoV-2 PsV Infection

The inhibition of SARS-CoV-2 PsV infection was determined by a single-cycle infection assay. Briefly, SARS-CoV-2 PsV was generated by cotransfecting HEK293T cells with a wild-type S protein-expressing plasmid and HIV-1 backbone plasmid (pNL4-3.Luc.RE) that encodes an Env-defective, luciferase reporter-expressing HIV-1 genome. Transfection was performed using polyethylenimine (PEI) at a 3:1 mass ratio (backbone: spike plasmid). Supernatants were harvested 48h post-transfection, filtered (0.45 μm), aliquoted, and stored at −80 °C. Caco-2 cells, which highly express TMPRSS2, were seeded into 96-well plates at a density of 2 × 10^4^ cells per well, 24 h prior to infection. Test samples were serially diluted from the stock solution, with a three-fold dilution performed seven times. Each diluted sample was added to Caco-2 cells and incubated at 37 °C for 1 h. The HIV-1-based pseudotyped virus incorporating SARS-CoV-2 spike protein was then added to the 96-well plates containing test compounds and Caco-2 (TMPRSS2^+^) cells. After 12 h of incubation, the medium was replaced with fresh culture medium, and the plates were further incubated at 37 °C for 48 h. The infection rate of the pseudovirus was subsequently determined by measuring luciferase activity using a luciferase detection system (Promega, Madison, WI, USA) and a multimode plate reader.

### 3.6. CD Spectroscopy

The bifunctional chimeras and N44 were separately dissolved in PBS (1×, pH 7.4) and dd H_2_O, respectively. The bifunctional chimeras were then incubated with an equimolar concentration of N44 at 37 °C for 30 min, with a final concentration of 10 µM. Circular dichroism (CD) spectra were recorded on a Chirascan Plus qCD instrument (Applied Photophysics, Leatherhead, UK) at room temperature, using a 0.1 nm step resolution over the wavelength range of 195 to 260 nm. The α-helical content was determined by normalizing the mean residue ellipticity [*θ*] at 222 nm to the value anticipated for complete helix formation (−33,000 deg cm^2^/dmol). Thermal denaturation experiments were conducted by monitoring the ellipticity changes at 222 nm as the temperature was increased from 20 °C to 90 °C at a rate of 2 °C/min.

### 3.7. Native-PAGE

Native-PAGE was conducted using 10% Tris-glycine gels and Tricine-glycine running buffer (pH 4.4). The bifunctional chimeras and N44 were separately dissolved in PBS (1×, pH 7.4) and dd H_2_O, respectively, to achieve a final concentration of 100 µM for each peptide. Equimolar mixtures of the bifunctional chimeras and N44 were prepared and incubated at 37 °C for 30 min at a final concentration of 100 µM. After incubation, the samples were mixed with Tris-glycine native sample buffer (4:1, *v*/*v*) and loaded onto the Native-PAGE gel (15 µL per well). Electrophoresis was carried out at room temperature for approximately 2 h at a constant voltage of 120 V. The gel was subsequently stained with Coomassie Blue R250 (Real-times Ltd., Beijing, China) and imaged using a ChampGel 6000 Imaging System (Sage Creation Ltd., Beijing, China).

### 3.8. Evaluation of Inhibition of TMPRSS2 Catalytic Activity

Test compounds were dissolved in DMSO and prepared as 1000 nmol/L stock solutions, which were then serially diluted nine times with a 1:4 concentration gradient. Using an Echo liquid handler, 0.1 µL of each compound was transferred into a 384-well reaction plate (Corning 4514), ensuring a final DMSO concentration of 1% (with two replicates per concentration). Subsequently, 5 µL of TMPRSS2 enzyme solution (working concentration: 5 nM) was added to each well, and the compounds were pre-incubated with the enzyme at 25 °C for 10 min. High control wells contained DMSO and TMPRSS2 enzyme, while low control wells contained DMSO and assay buffer (25 mM Tris, 75 mM NaCl, 2 mM CaCl_2_, pH 8.0). Next, 5 µL of Boc-Gln-Ala-Arg-AMC substrate solution (working concentration: 100 µM) was added to each well, followed by incubation at 25 °C for 60 min. Fluorescence signals (Ex: 390 nm/Em: 460 nm) were measured using a BMG plate reader. The results were compared with the controls to calculate the inhibition rates, and the IC_50_ values were determined by fitting the data using GraphPad Prism 8 software.

### 3.9. SPR Analysis

SPR analysis was performed on a Biacore 8K instrument (GE Healthcare, IL, USA). TMPRSS2 was covalently attached to a CM5 sensor chip via amine coupling chemistry, achieving a final immobilization level of ~3000 resonance units (RU) under a continuous flow rate of 10 μL/min. Serial dilutions of chimeras were injected across the sensor surface for 60 s of association, followed by a 60 s dissociation phase at 30 μL/min. Background binding to blank immobilized flow cells was subtracted. Binding affinity (*K_D_*) was determined using a 1:1 binding steady-state model built in Biacore 8K Evaluation Software.

## 4. Conclusions

In summary, dual-targeted entry inhibitors IP4X and IP4Z, formed by covalently linking a TMPRSS2 inhibitor, Camostat, and an HR1-targeting peptide fusion inhibitor IPB19 via a PEG4 linker, showed potent inhibitory activities against SARS-CoV-2, including the Omicron variant. We demonstrated that compounds IP4X and IP4Z presented a dual mechanism of interacting with both TMPRSS2 and the HR1 region of the S protein. These findings are promising for the further development of bifunctional entry inhibitors as novel anti-SARS-CoV-2 drugs and enrich the understanding of S-mediated entry of SARS-CoV-2 into host cells.

## Figures and Tables

**Table 1:** Antiviral activity of entry inhibitors against SARS-CoV-2 pseudovirus ^a^.

Name	Sequence	IC_50_ (µM)
Class I chimeras		
LP4X	CmX-PEG4--DKEWILQKIYELMRILDELGHAEASMRVEDLIYEFMK-CONH_2_	0.40 ± 0.06
LP4Z	CmZ-PEG4--DKEWILQKIYELMRILDELGHAEASMRVEDLIYEFMK-CONH_2_	0.45 ± 0.20
LP24X	CmX-PEG24-DKEWILQKIYELMRILDELGHAEASMRVEDLIYEFMK-CONH_2_	0.32 ± 0.03
LP24Z	CmZ-PEG24-DKEWILQKIYELMRILDELGHAEASMRVEDLIYEFMK-CONH_2_	0.33 ± 0.01
Class II chimeras		
IP4X	CmX-PEG4--SVVNIQKEIDRLNEVAKNLNESLIDLQELGKYEQYIK-CONH_2_	0.16 ± 0.07
IP4Z	CmZ-PEG4--SVVNIQKEIDRLNEVAKNLNESLIDLQELGKYEQYIK-CONH_2_	0.17 ± 0.09
IP24X	CmX-PEG24-SVVNIQKEIDRLNEVAKNLNESLIDLQELGKYEQYIK-CONH_2_	0.29 ± 0.10
IP24Z	CmZ-PEG24-SVVNIQKEIDRLNEVAKNLNESLIDLQELGKYEQYIK-CONH_2_	0.32 ± 0.18
Control compounds		
LCB1T	Ac-DKEWILQKIYELMRILDELGHAEASMRVEDLIYEFMK-CONH_2_	0.44 ± 0.04
IPB19	Ac-SVVNIQKEIDRLNEVAKNLNESLIDLQELGKYEQYIK-CONH_2_	2.43 ± 0.61
Camostat	-	4.60 ± 2.32

^a^ IC_50_ values were obtained with wild-type SARS-CoV-2 PsV in Caco2 cells that express TMPRSS2 on the surface. The assay was performed in three independent experiments (one independent experiment of three is shown), and the data are expressed as the mean ± standard deviation.

## Data Availability

The original contributions presented in this study are included in the article. Further inquiries can be directed to the corresponding authors.

## Supplementary Materials

The following supporting information can be downloaded at https://www.mdpi.com/article/10.3390/ijms26178289/s1.
