# Supplementary material for: A Bifunctional SARS-CoV-2 Entry Inhibitor Targeting the Host Protease TMPRSS2 and Viral Spike Protein HR1 Region
Source: Int J Mol Sci. 2025 Aug 26;26(17):8289. doi: 10.3390/ijms26178289 (PMC12428505; doi:10.3390/ijms26178289)
Supplement: Supplementary file 1 [file ijms-26-08289-s001.zip › ijms-3720114-supplementary.pdf]

Supplementary Data

A Bifunctional SARS-CoV-2 Entry Inhibitor Targeting the Host Protease TMPRSS2 and Viral Spike Protein HR1 Region

Huan Wang,[a,¶] Qing Li,[a,¶] Zhe Yin,[b,¶], Shu Du,[a] Longbo Zheng,[a,c] Xinmeng Du,[a,c] Anqi Shi,[d] Jichun Li,[a] Weiguo Shi,[a] Fei Yu,[b] Junhai Xiao,[*,a] Chao Wang,[*,a]

[a] State Key Laboratory of National Security Specially Needed Medicines, Beijing Institute of Pharmacology and Toxicology, Beijing 100850, China;
[b] Hebei Key Laboratory of Analysis and Control of Zoonotic Pathogenic Microorganism, College of Life Sciences, Hebei Agricultural University, Baoding 071001, China;
[c] Key Laboratory of Structure-based Drug Design & Discovery of the Ministry of Education, Shenyang Pharmaceutical University, Shenyang 110016, China;
[d] College of Pharmacy, Mudanjiang Medical University, Mudanjiang 157000, China
[¶]These authors contributed equally to this work.

Corresponding Authors:

C.W.: State Key Laboratory of Toxicology and Medical Countermeasures, Beijing Institute of Pharmacology & Toxicology, 27 Tai-Ping Road, Beijing 100850, China; Tel.: 86-10-6693-0695; Fax: 86-10-6821-1656, E-mail: chaow301@sina.com.

J.X.: State Key Laboratory of Toxicology and Medical Countermeasures, Beijing Institute of Pharmacology & Toxicology, 27 Tai-Ping Road, Beijing 100850, China; Tel.: 86-10-6693-0695, E-mail: xiaojunhai@139.com.

Table of Contents

Supplemental Tables and Figures

Table S1. HPLC method used for the purification of peptide compounds………….……………… S2
Table S2. HPLC method used for the analysis of peptide compounds……………..…………… S2
Table S3. Antiviral activity of entry inhibitors against pseudotyped SARS-CoV-2 Omicron strain.S2
Figure S1. MALDI-TOF-MS and analytical HPLC of designed peptides………..……………… S3

Table S1. HPLC method used for the purification of peptide compounds[a]

Time (min)	Solvent A (%)	Solvent B (%)
5	60	40
25	50	50
50	35	65
60	35	65
65	60	40

[a] Crude peptide products were purified by preparative reverse-phase HPLC with a Shimadzu preparative HPLC system (LC-20A) on a Thermo Scientific HYPERSIL PREP HS C8 column (30mm × 250mm, 5μm) at constant flow rate of 15 mL/min. Solvent A: 0.1% trifluoroacetic acid in H_2O; Solvent B: 0.1% trifluoroacetic acid in 70% CH_3CN/H_2O.

Table S2. HPLC method used for the analysis of peptide compounds[a]

Methods	Time (min)	Solvent A (%)	Solvent B (%)
Method A	5	50	50
	10	0	100
	15	0	100
	20	0	100
	23	0	100
	25	90	10
Method B	5	50	50
	10	30	70
	15	10	90
	20	0	100
	34	0	100
	35	90	10

[a] Peptide compounds were analyzed by analytical RP-HPLC performed on a RP-C8 column (X-Bridge C8, 4.6 × 250 mm, 5 μm) using two different solvent systems (Methods A and B) and a flow rate of 1 mL/min with detection at 210 nm. Solvent A: 0.1% trifluoroacetic acid in H_2O; Solvent B: 0.1% trifluoroacetic acid in 70% CH_3CN/H_2O.

Table S3. Antiviral activity of entry inhibitors against pseudotyped SARS-CoV-2 Omicron strain [a]

Name	IC_{50} (μM)
IP4X	0.83±0.01
IP4Z	0.76±0.08

[a] IC_{50} values were obtained with pseudotyped SARS-CoV-2 Omicron strain in Caco2 cells that express TMPRSS2 on the surface. The assay was performed in triplicate, and the data are expressed as the mean ± standard deviation.

MALDI-TOF-MS and analytical HPLC of designed peptides

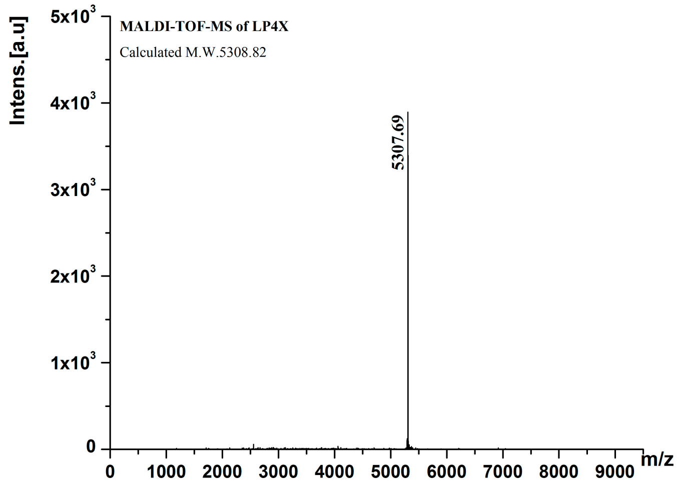

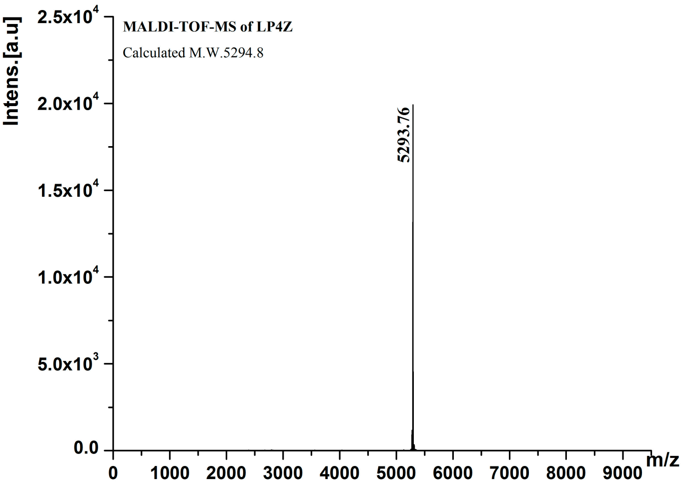

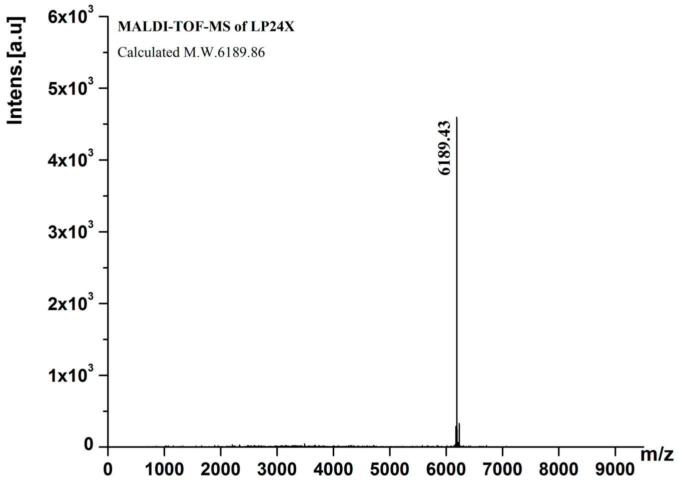

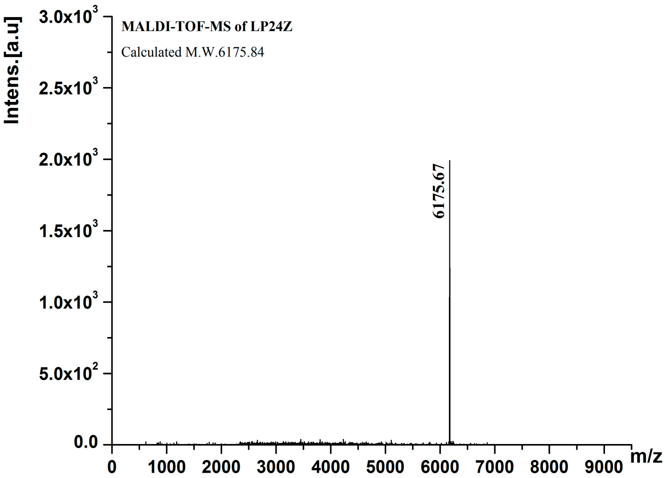

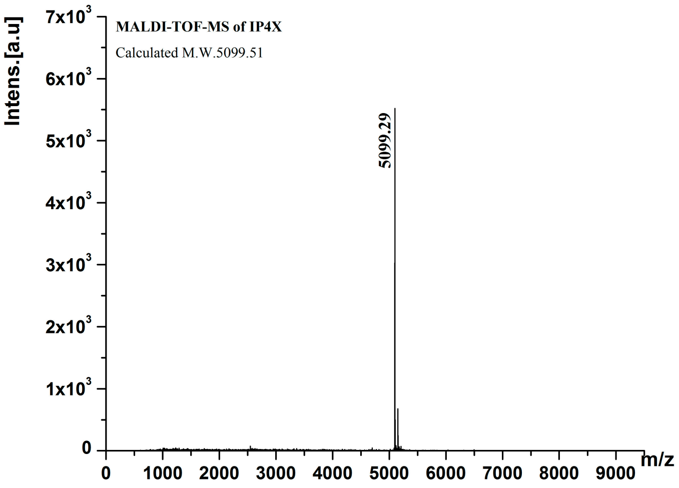

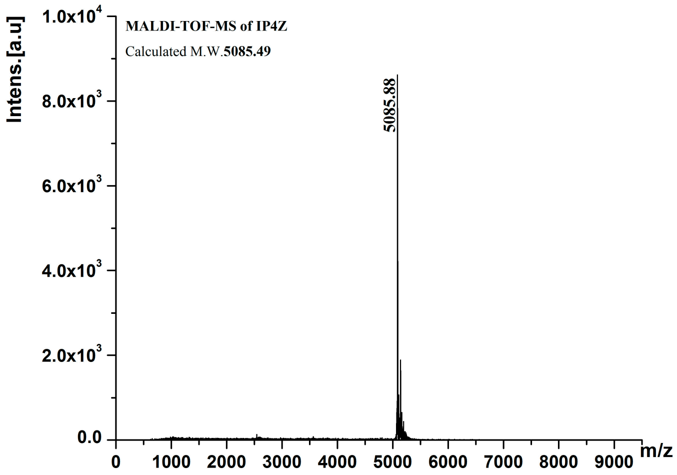

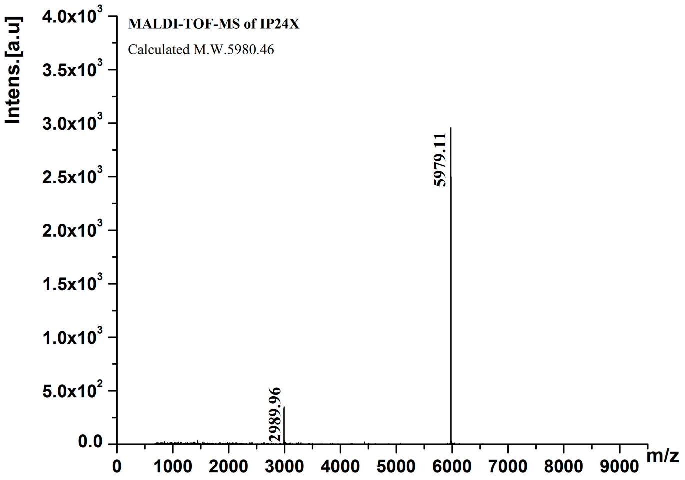
MALDI-TOF-MS of IP24X
Calculated M.W.5980.46
5979.11
2989.96
Intens.[a.u]
m/z

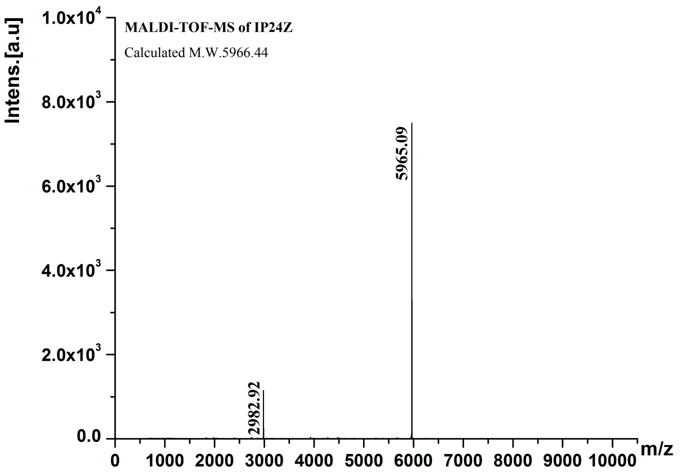
MALDI-TOF-MS of IP24Z
Calculated M.W.5966.44
5965.09
2982.92
Intens.[a.u]
m/z

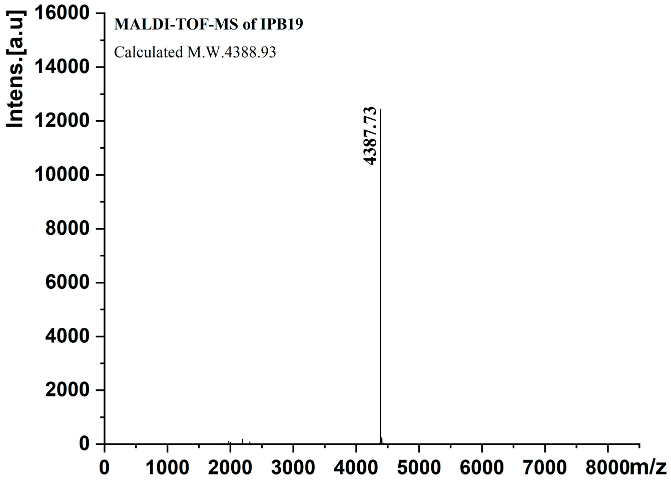
MALDI-TOF-MS of IPB19
Calculated M.W.4388.93
4387.73
Intens.[a.u]
m/z

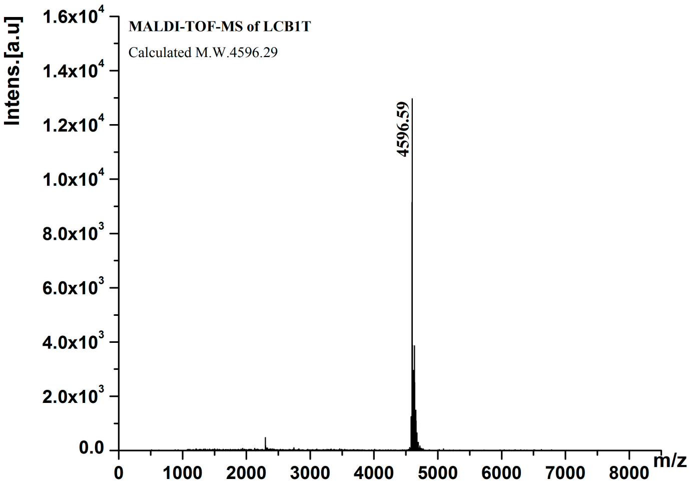
MALDI-TOF-MS of LCB1T
Calculated M.W.4596.29
4596.59
Intens.[a.u]
m/z

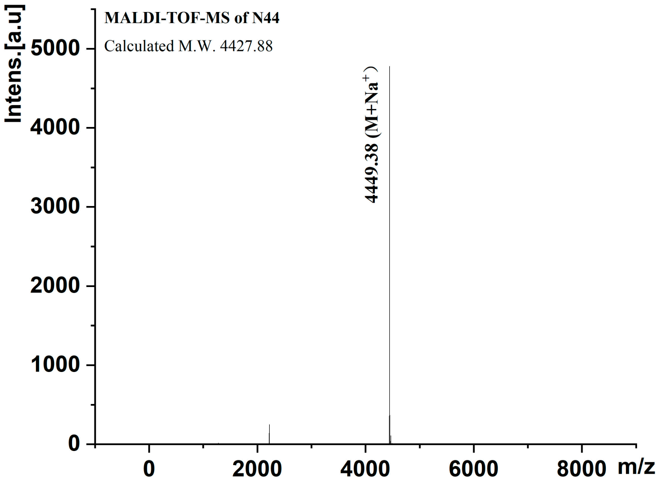
MALDI-TOF-MS of N44
Calculated M.W. 4427.88
4449.38 (M+Na+)
Intens.[a.u]
m/z

Analytical HPLC of LP4X
[98 % purity, t_R (Method A) = 12.83 min]

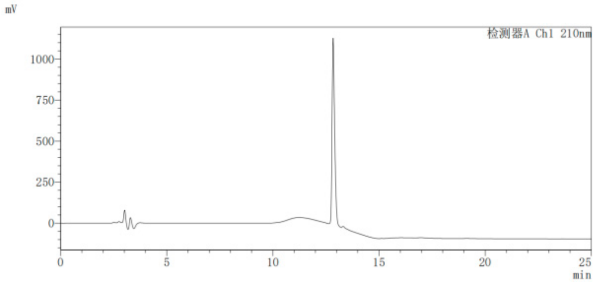

Analytical HPLC of LP4X
[98 % purity, t_R (Method B) = 16.14 min]

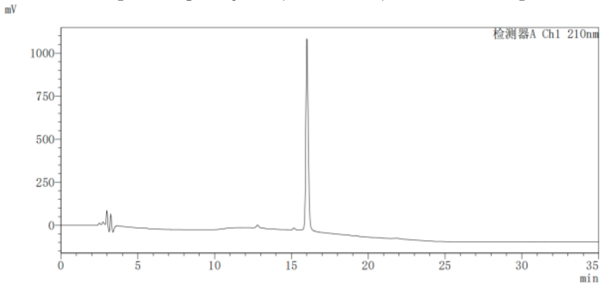

Analytical HPLC of LP4Z
[99 % purity, t_R (Method A) = 13.36 min]

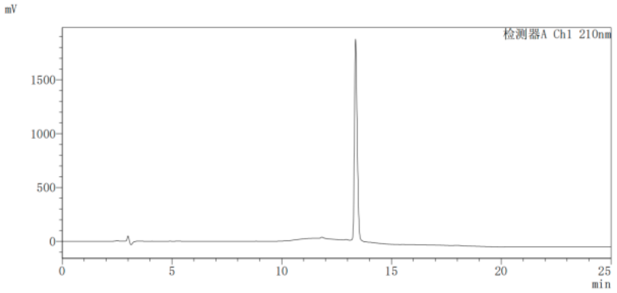

Analytical HPLC of LP4Z
[98 % purity, t_R (Method B) = 15.99 min]

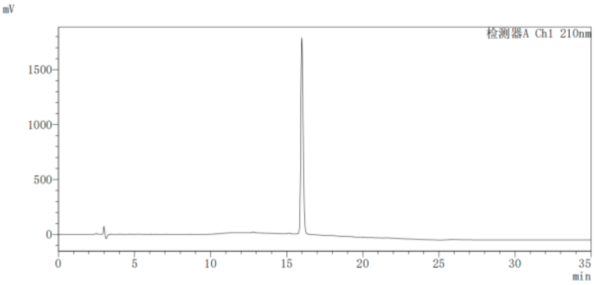

Analytical HPLC of LP24X
[99 % purity, t_R (Method A) = 13.84 min]

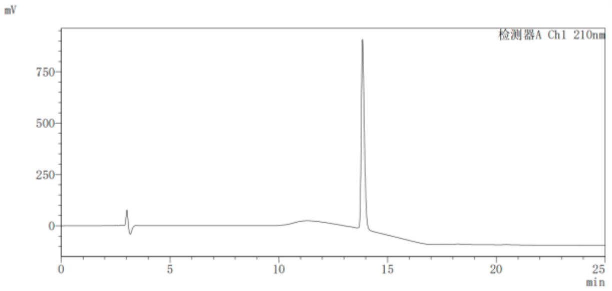

Analytical HPLC of LP24X
[99 % purity, t_R (Method B) = 16.27 min]

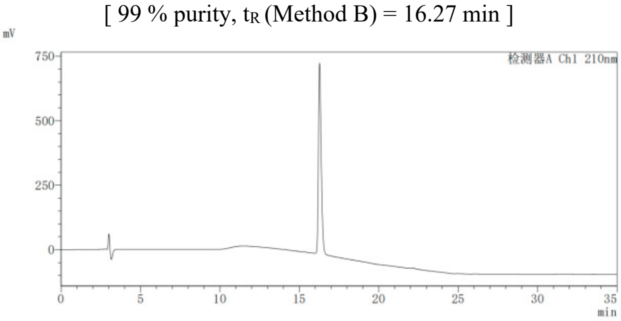

Analytical HPLC of LP24Z
[99 % purity, t_R (Method A) = 13.83 min]

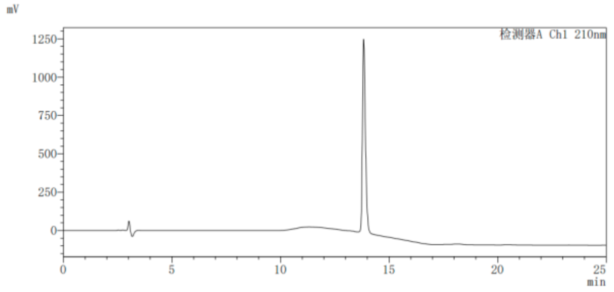

Analytical HPLC of LP24Z
[99 % purity, t_R (Method B) = 16.14 min]

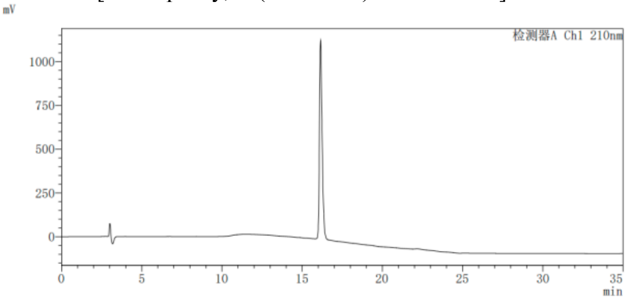

Analytical HPLC of IP4X
[99 % purity, t_R (Method A) = 11.63 min]

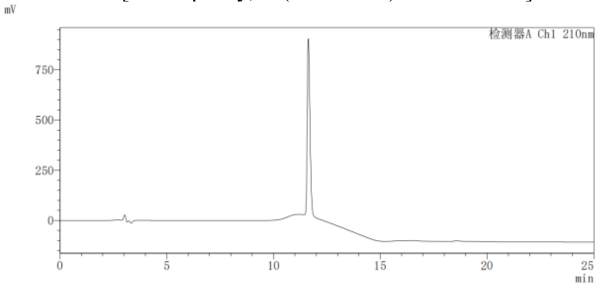

Analytical HPLC of IP4X
[98 % purity, t_R (Method B) = 12.88 min]

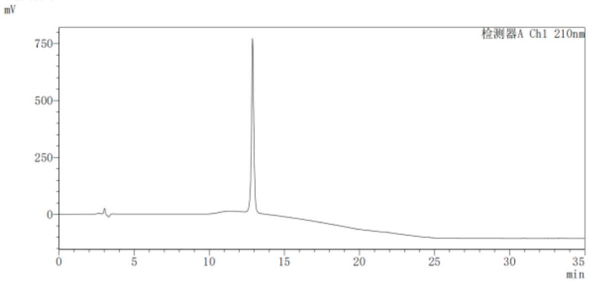

Analytical HPLC of IP4Z
[99 % purity, t_R (Method A) = 11.91 min]

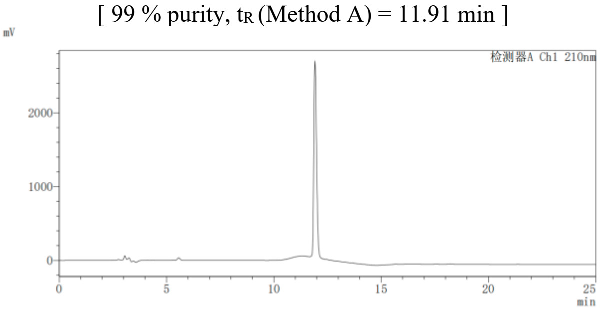

Analytical HPLC of IP4Z
[98.7 % purity, t_R (Method B) = 13.77 min]

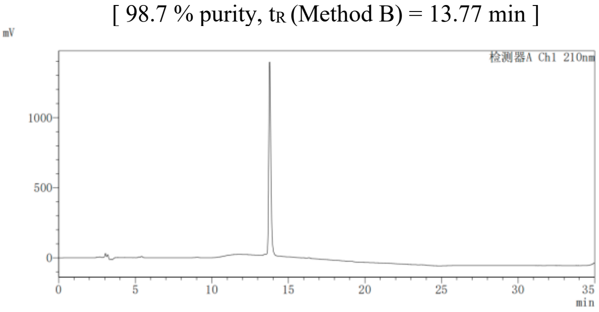

Analytical HPLC of IP24X
[99 % purity, t_R (Method A) =11.82 min]

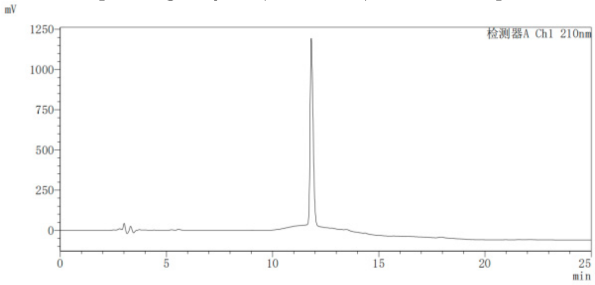

Analytical HPLC of IP24X
[99 % purity, t_R (Method B) = 12.73 min]

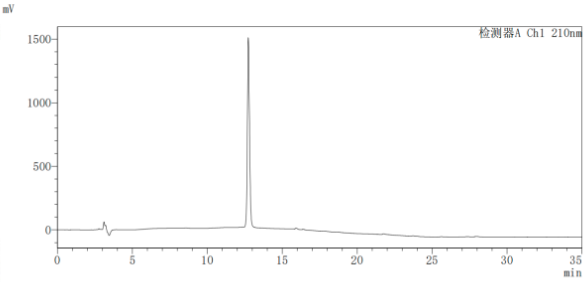

Analytical HPLC of IP24Z
[99 % purity, t_R (Method A) = 11.87 min]

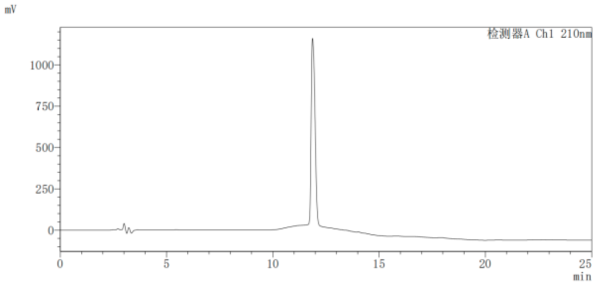

Analytical HPLC of IP24Z
[99 % purity, t_R (Method B) = 12.86 min]

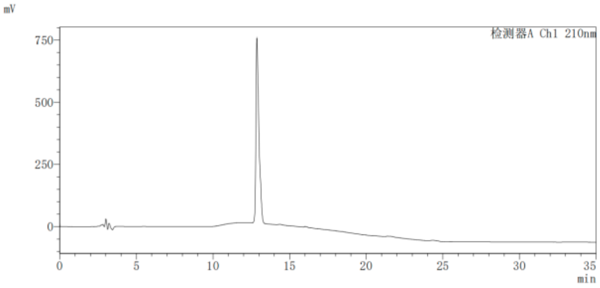

Analytical HPLC of IPB19
[99 % purity, t_R (Method A) = 11.77 min]

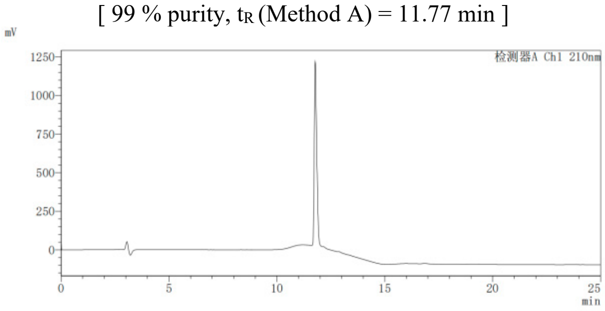

Analytical HPLC of IPB19
[99 % purity, t_R (Method B) = 13.20 min]

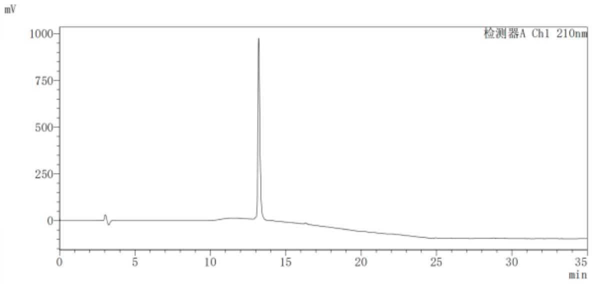

Analytical HPLC of LCB1T
[99 % purity, t_R (Method A) = 13.17 min]

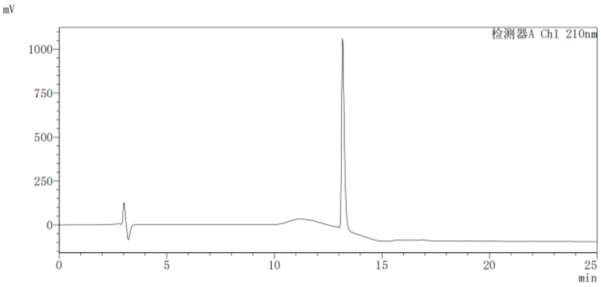

Analytical HPLC of LCB1T
[97 % purity, t_R (Method B) = 16.94 min]

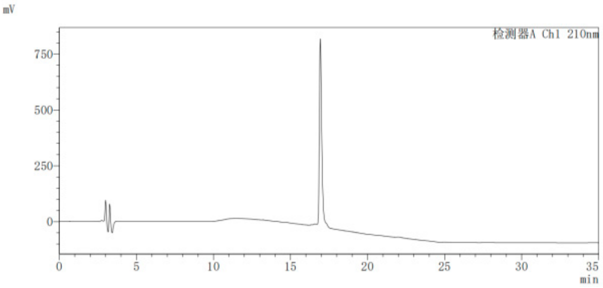

Analytical HPLC of N44
[97 % purity, t_R (Method A) = 14.38 min]

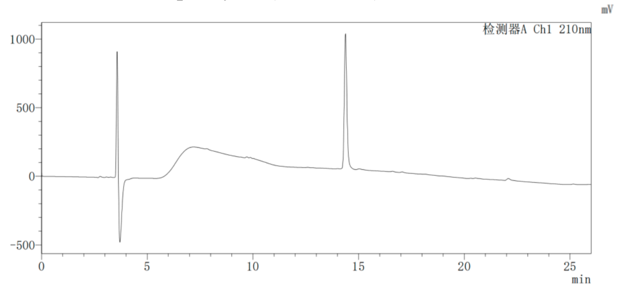

Analytical HPLC of N44
[98 % purity, t_R (Method B) = 16.21 min]

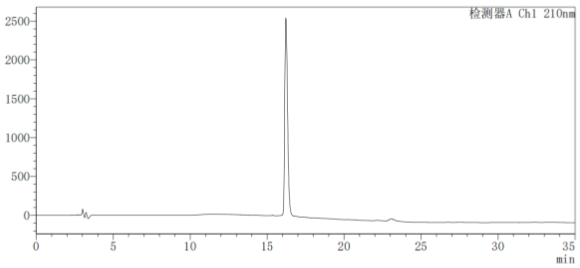

Figure S1. MALDI-TOF-MS and Analytical HPLC of designed peptides
